# Renal artery denervation prevents ventricular arrhythmias in long QT rabbit models

**DOI:** 10.1038/s41598-022-06882-5

**Published:** 2022-02-21

**Authors:** An Nu-Khanh Ton, Shin-Huei Liu, Li-Wei Lo, Thien Chuong-Nguyen Khac, Yu-Hui Chou, Wen-Han Cheng, Wei-Lun Lin, Tzu-Yen Peng, Pin-Yi Lin, Shih-Lin Chang, Shih-Ann Chen

**Affiliations:** 1 Division of Cardiology, Department of Medicine, Heart Rhythm Center, Taipei Veterans General Hospital, No. 201, Sec. 2, Shih-Pai Road, Taipei, Taiwan; 2 Institute of Clinical Medicine and Cardiovascular Research Institute, National Yang Ming Chiao Tung University, Taipei, Taiwan; 3 Faculty of Medicine, School of Medicine, National Yang Ming Chiao Tung University, Taipei, Taiwan; 4 Institute of Biomedical Sciences, Mackay Medical College, New Taipei City, Taiwan; 5 Cardiovascular Center, Taichung Veterans General Hospital, Taichung, Taiwan

**Keywords:** Neuroscience, Cardiology

## Abstract

Long QT syndrome (LQTS) is commonly presented with life-threatening ventricular arrhythmias (VA). Renal artery denervation (RDN) is an alternative antiadrenergic treatment that attenuates sympathetic activity. We aimed to evaluate the efficacy of RDN on preventing VAs in LQTS rabbits induced by drugs. The subtypes of LQTS were induced by infusion of HMR-1556 for LQTS type 1 (LQT1), erythromycin for LQTS type 2 (LQT2), and veratridine for LQTS type 3 (LQT3). Forty-four rabbits were randomized into the LQT1, LQT2, LQT3, LQT1-RDN, LQT2-RDN, and LQT3-RDN groups. All rabbits underwent cardiac electrophysiology studies. The QTc interval of the LQT2-RDN group was significantly shorter than those in the LQT2 group (650.08 ± 472.67 vs. 401.78 ± 42.91 ms, *p* = 0.011). The QTc interval of the LQT3-RDN group was significantly shorter than those in the LQT3 group (372.00 ± 22.41 vs. 335.70 ± 28.21 ms, *p* = 0.035). The VA inducibility in all subtypes of the LQT-RDN groups was significantly lower than those in the LQT-RDN groups, respectively (LQT1: 9.00 ± 3.30 vs. 47.44 ± 4.21%, *p* < 0.001; LQT2: 11.43 ± 6.37 vs. 45.38 ± 5.29%, *p* = 0.026; LQT3: 10.00 ± 6.32 vs. 32.40 ± 7.19%, *p* = 0.006). This study demonstrated the neuromodulation of RDN leading to electrical remodeling and reduced VA inducibility of the ventricular substrate in LQT models.

## Introduction

Congenital long QT syndrome (LQTS) is a congenital cardiac disorder that prolongs ventricular repolarization leading to ventricular arrhythmias (VAs) or sudden cardiac death (SCD). Beta-blocking (BB) agents are the cornerstone in preventing cardiovascular (CV) events in LQTS, however, 32% of symptomatic VAs and 14% of cardiac arrests were identified in a 5-year follow-up^1^. The implanted cardioverter defibrillator (ICD) reduces the incidence of SCD but cannot prevent the occurrence of life-threatening VAs. Therefore, the demand for an alternative approach is required for drug-refractory LQTS patients. Recently, neuromodulation such as left cardiac sympathetic denervation (LCSD) successfully reduced the VAs and SCD^2,3^. However, 23% of symptomatic VAs and 95% of LCSD-related adverse effects were documented^4,5^. Alternatively, renal artery denervation (RDN) is another effective neuromodulation with favorable results in suppressing life-threatening VAs^6–8^. Previous studies have demonstrated the efficacy of RDN, including reduced VA occurrence in electrical storms, prevention of VAs in heart failure (HF), and myocardial infarction subjects^9–11^. Despite favorable results from previous research, the exact role and mechanism of RDN in LQTS remained questionable. Finally, the similar ion channels between rabbit and human hearts were demonstrated in previous research was the reason for choosing a rabbit model many LQTS research. We aimed to investigate the cardiac electrophysiological (EP) properties and arrhythmogenicity of RDN as an alternative approach to prevent VAs in the LQTS rabbit model.

## Results

### SR CL and QTc interval on ECG

**Figure 1:**
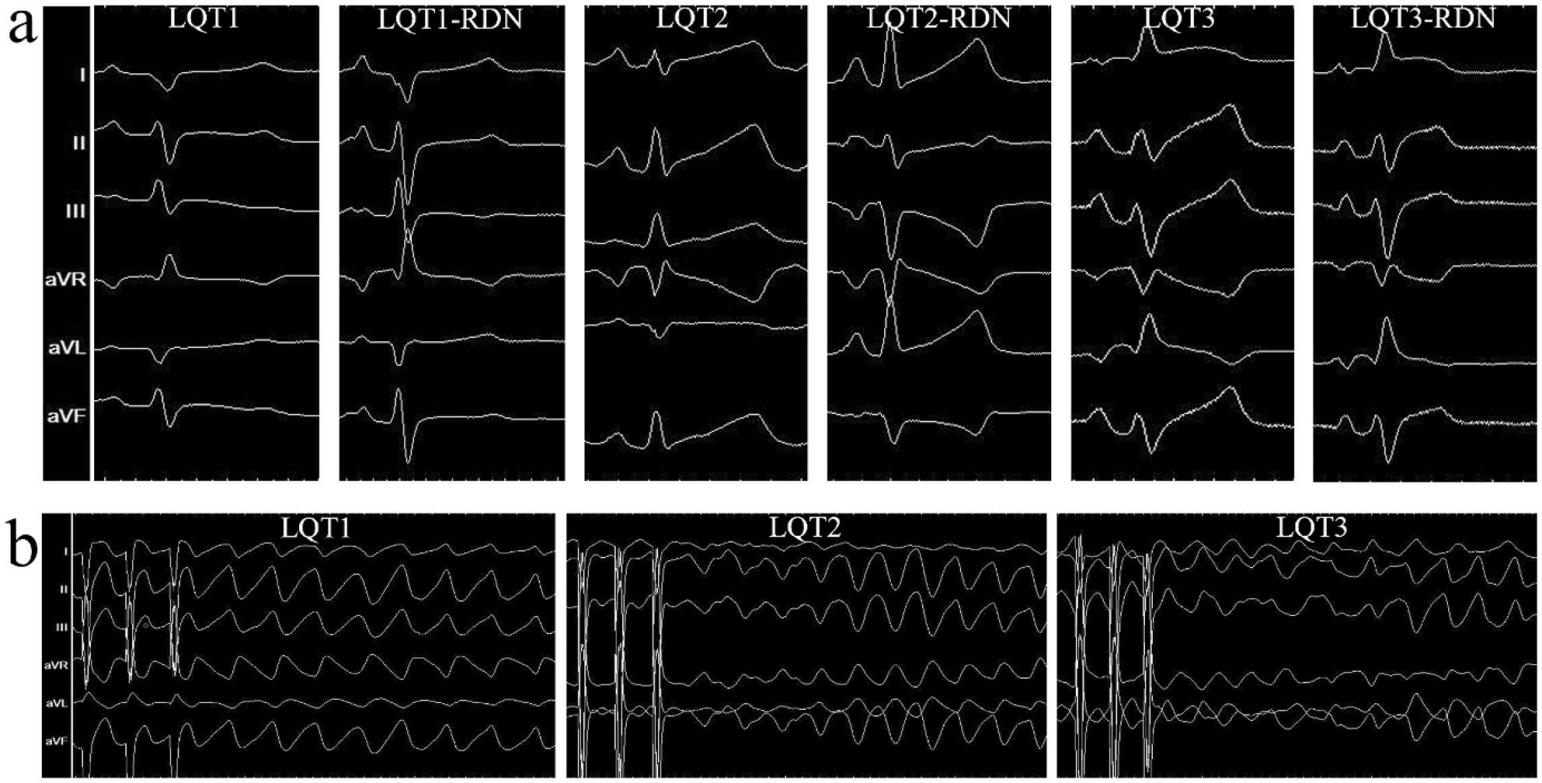
(**a**) Representative examples of ECG under the final LQTS induction dose in all groups. (**b**) Representative examples of ECG of inducible VAs in the LQT1, LQT2, and LQT3 groups.

Table 1 demonstrated the sinus rhythm (SR) cycle length (CL) measurements from the baseline to the sequentially increased doses for LQT subtype induction. In the LQT1 and LQT1-RDN groups, the SR CL gradually prolonged under sequential doses of HMR-1556 when compared with those at the baseline within each group (Table 1). Similar results of SR CL prolongation were found in the LQT2, LQT2-RDN, LQT3, and LQT3-RDN groups under sequential doses of LQT induction when compared to those at the baseline within each group (Table 1). Under the same LQT subtype induction dose, the SR CL between the LQT and LQT-RDN groups showed no difference in all the LQT subtypes (LQT1, LQT2, and LQT3) (Table 1). Figure 1a illustrated representative examples of ECG under the final LQTS induction dose in all groups. Figure 1b illustrated inducible VAs in the LQT1, LQT2, and LQT3 groups. Table 2 demonstrated the QTc interval measurements from the baseline to the sequentially increased drug doses for LQT subtype induction. In the LQT1 and LQT1-RDN groups, the QTc intervals were gradually prolonged under sequential doses of HMR-1556 when compared with those at the baseline within each group (Table 2). Similar results of QTc interval prolongation were found in the LQT2, LQT2-RDN, LQT3, and LQT3-RDN groups under sequentially increased drug doses of LQT induction when compared to those at the baseline within each group (Table 2). Under the same LQT subtype induction dose, the QTc intervals between the LQT and LQT-RDN groups showed no difference in all the LQT subtypes (LQT1, LQT2, and LQT3) (Table 2).

**Table 1:** The SR CL (ms) measurements from baseline to the sequentially increased doses of all LQTS subtype induction (LQTS induction drugs and doses are described in the “Methods” section).

	LQT1	LQT1-RDN	P	LQT2	LQT2-RDN	P	LQT3	LQT3-RDN	P
Baseline	349.67 ± 29.13	344.56 ± 41.85	0.768	353.50 ± 55.60	356.57 ± 32.61	0.901	319.00 ± 12.21	317.83 ± 43.32	0.955
1st Dose	379.44 ± 53.79	376.67 ± 41.14	0.904	395.88 ± 85.92	383.71 ± 34.12	0.732	339.60 ± 18.99	348.17 ± 40.65	0.677
2nd Dose	373.01 ± 50.67	383.05 ± 52.55	0.687	417.86 ± 40.09	429.00 ± 57.13	0.680	331.20 ± 23.77	356.00 ± 63.40	0.432
3rd Dose	399.67 ± 45.79	383.56 ± 56.55	0.516	474.25 ± 85.13	496.00 ± 67.49	0.774	346.20 ± 35.22	357.50 ± 42.41	0.647
4th Dose	395.33 ± 40.07	391.56 ± 55.40	0.870	–	–	–	336.00 ± 41.95	365.33 ± 54.42	0.351

**Table 2:** The QTc interval measurement (ms) from baseline to the sequentially increased doses of all LQTS subtype induction (LQTS induction drugs and doses are described in the “Methods” section).

	LQT1	LQT1-RDN	P	LQT2	LQT2-RDN	P	LQT3	LQT3-RDN	P
Baseline	284.90 ± 15.82	289.55 ± 14.07	0.102	317.24 ± 22.88	314.66 ± 17.22	0.052	298.25 ± 16.40	331.11 ± 38.72	0.071
1st Dose	292.30 ± 14.50	302.20 ± 7.66	0.089	347.43 ± 34.74	325.50 ± 27.52	0.203	311.53 ± 32.78	325.65 ± 40.42	0.312
2nd Dose	303.37 ± 18.21	306.74 ± 23.26	0.193	391.84 ± 44.14	355.62 ± 22.41	0.042	337.67 ± 15.54	325.73 ± 50.81	0.628
3rd Dose	308.42 ± 15.77	313.35 ± 18.63	0.154	650.08 ± 472.67	401.78 ± 42.91	0.011	363.84 ± 9.40	328.59 ± 30.23	0.046
4th Dose	321.26 ± 15.90	338.81 ± 27.02	0.113	–	–	–	372.00 ± 22.41	335.70 ± 28.21	0.035

### Cardiac EP study and VA inducibility test

**Figure 2:**
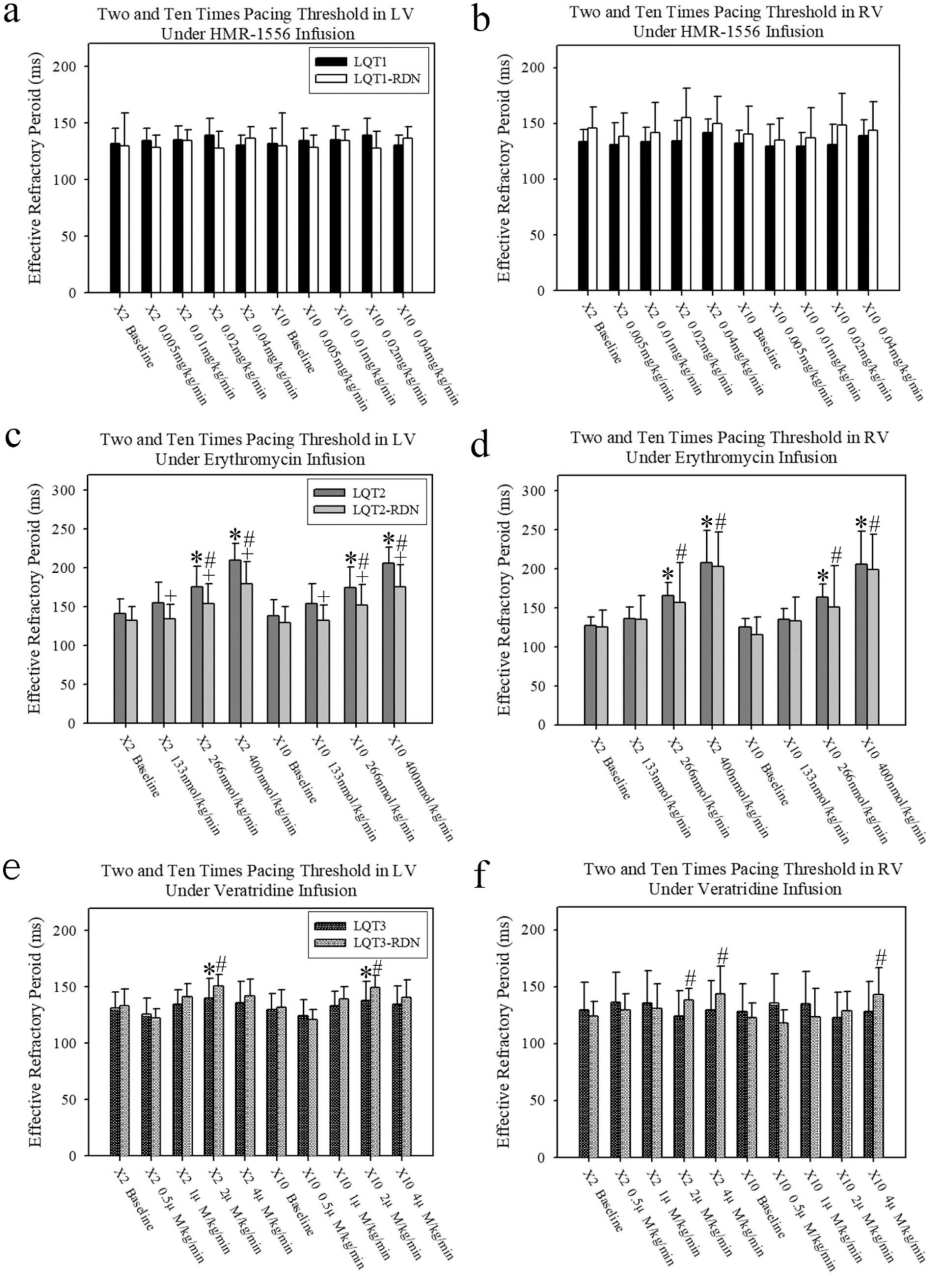
The ERPs at 2 and 10 times pacing threshold in the LV and RV under LQTS subtype induction. (**a**) The ERPs at 2 and 10 times pacing threshold in the LV under HMR-1556 infusion. (**b**) The ERPs at 2 and 10 times pacing threshold in the RV under HMR-1556 infusion. (**c**) The ERPs at 2 and 10 times pacing threshold in the LV under erythromycin infusion. (**d**) The ERPs at 2 and 10 times pacing threshold in the RV under erythromycin infusion. (**e**) The ERPs at 2 and 10 times pacing threshold in the LV under veratridine infusion. (**f**) The ERPs at 2 and 10 times pacing threshold in the RV under veratridine infusion. (*p < 0.05 compared with baseline within the LQT group. #*p* < 0.05 compared with baseline within the LQT-RDN group. +*p* < 0.05 compared between the LQT and LQT-RDN groups under the same concentration of LQTS drug infusion).

**Figure 3:**
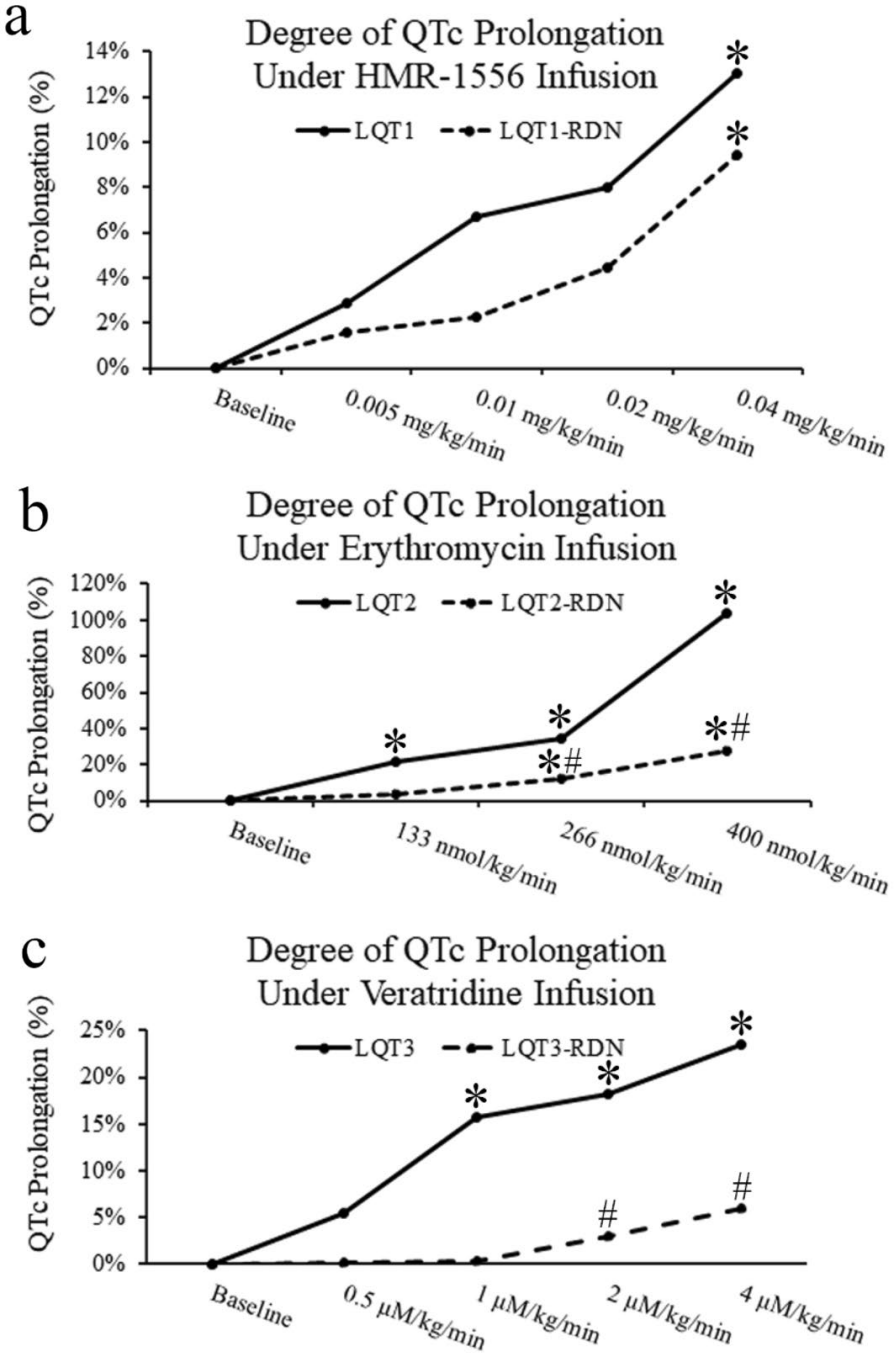
Degree of QTc interval prolongation under LQTS induction. (**a**) QTc prolongation of the LQT1 and LQT1-RDN groups under sequential HMR-1556 infusion. (**b**) QTc prolongation of the LQT2 and LQT2-RDN groups under sequential erythromycin infusion. (**c**) QTc prolongation of the LQ3 and LQT3-RDN groups under sequential veratridine infusion. (*p < 0.05 compared with baseline within the same group. #*p* < 0.05 compared between LQT and LQT-RDN groups under the same concentration of LQTS drug infusion).

Figure 2 demonstrated the effective refractory periods (ERP) of LV and RV at 2 times and 10 times pacing threshold under sequentially increased drug doses of LQT induction in each group, respectively. In LV and RV, the ERPs during 2 times and 10 times pacing threshold revealed no difference between the baseline and those with sequentially increased HMR-1556 dose within the LQT1 and LQT1-RDN groups, respectively (Fig. 2a, b). In LV and RV, the ERPs during 2 times and 10 times pacing threshold revealed no difference between the LQT1 and LQT1-RDN groups at baseline and each HMR-1556 doses, respectively (Fig. 2a, b). Figure 3a demonstrated the percentages of QTc interval prolongation in the LQT1 and LQT1-RDN groups under sequential HMR-1556 infusion. Under HMR-1556 0.04 mg/kg/min infusion, the percentages of QTc interval prolongation in the LQT1 and LQT1-RDN groups were significantly longer than those in the baseline, respectively (Fig. 3a).

In LV and RV, the ERPs during 2 times and 10 times pacing threshold gradually prolonged under sequentially increased erythromycin doses in the LQT2 and LQT2-RDN groups with significant difference between those at the baseline and final erythromycin doses within each group, respectively (Fig. 2c, d). In LV, the ERPs during 2 times and 10 times pacing threshold of the LQT2-RDN groups in each erythromycin dose were significantly shorter than those in the LQT2 groups, respectively (Fig. 2c). Figure 3b demonstrated the percentage of QTc interval prolongation in the LQT2 and LQT2-RDN groups under sequential erythromycin infusion. Under erythromycin 133, 266, and 400 nmol/kg/min infusion, the percentages of QTc interval prolongation in the LQT2 group were significantly longer than those in the baseline, respectively (Fig. 3b). Under erythromycin 266 and 400 nmol/kg/min infusion, the percentages of QTc interval prolongation in the LQT2-RDN group were significantly longer than those in the baseline, respectively (Fig. 3b). Under erythromycin 266 and 400 nmol/kg/min infusion, the percentages of QTc interval prolongation in the LQT2 group were significantly longer than those in the LQT2-RDN group, respectively (Fig. 3b).

In LV, the ERPs during 2 times and 10 times pacing threshold gradually prolonged under sequentially increased veratridine doses in the LQT3 and LQT3-RDN groups with significant difference between those at the baseline and veratridine 2 µM/kg/min infusion within each group, respectively (Fig. 2e). In RV, the ERPs during 2 times and 10 times pacing threshold gradually prolonged under sequentially increased veratridine doses in the LQT3 and LQT3-RDN groups, respectively (Fig. 2f). In RV, the ERPs during 2 times and 10 times pacing threshold revealed a significant difference between those at the baseline and veratridine 4 µM/kg/min infusion within the LQT3-RDN group, respectively (Fig. 2f). Figure 3c demonstrated the percentage of QTc interval prolongation in the LQT3 and LQT3-RDN groups under sequential veratridine infusion. Under veratridine 1, 2, and 4 µM/kg/min infusion, the percentages of QTc interval prolongation in the LQT3 group were significantly longer than those in the baseline, respectively (Fig. 3c). Under veratridine 2 and 4 µM/kg/min infusion, the percentages of QTc interval prolongation in the LQT3-RDN group were significantly longer than those in the baseline, respectively (Fig. 3c).

**Figure 4:**
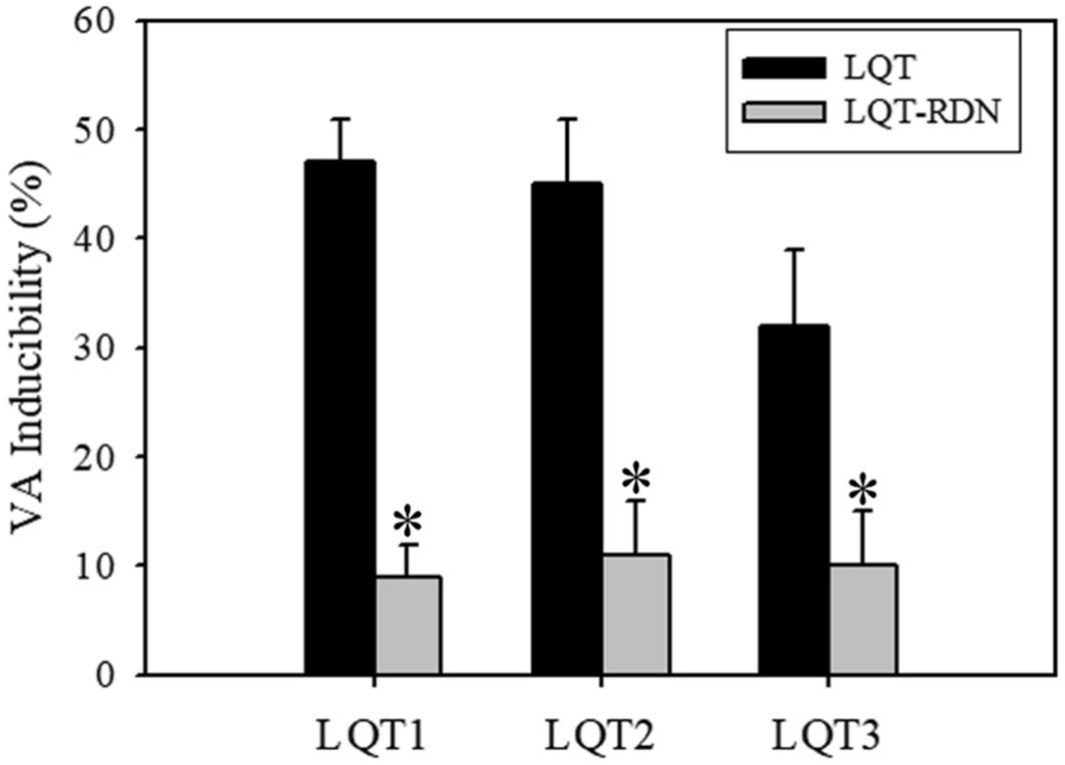
The VA inducibility test of all LQTS subtypes (LQT1, LQT2, LQT3) between the LQT and LQT-RDN groups. (*p < 0.05 compared with the LQT group in each subtype).

Two rabbits in the LQT1 group developed spontaneous VAs at baseline, whereas the LQT1-RDN group revealed no spontaneous arrhythmias. The VAs inducibility in the LQT1-RDN group was significantly lower than in the LQT1 group (Fig. 4). Two rabbits in the LQT2 group developed spontaneous VAs, whereas 1 rabbit in the LQT2-RDN group developed spontaneous VA. The VA inducibility in the LQT2-RDN group was significantly lower than in the LQT2 group (Fig. 4). Finally, there were no documented spontaneous VAs in both the LQT3 and LQT3-RDN groups. The VA inducibility in the LQT3-RDN group was significantly lower than in the LQT3 group (Fig. 4).

## Discussion

The main findings of this study are 1. The QTc interval prolongations were less prominent in the LQT-RDN groups than those in the LQT groups after LQT subtype induction; 2. The RDN decreased the burden of spontaneous and inducible VAs. Neuromodulation via RDN potentially prevented VAs through electrical remodeling in the ventricular substrate. These findings suggested RDN as a potential alternative therapeutic strategy for drug-refractory LQTS patients. Previous investigations revealed shortened QTc intervals after neuromodulation of LCSD in patients with LQTS and catecholaminergic polymorphic VAs^3^. Schwartz et al. demonstrated the LCSD method efficiently shortens the QTc interval in a 3-month follow-up and maintenance of QTc interval < 500 ms predicted a favorable outcome in patients who underwent the LCSD procedure^2,12^. These studies demonstrated neuromodulation via interventional antiadrenergic treatments lead to electrical remodeling and myocardial substrate modification^2^. In our study, the RDN reversed QTc interval prolongation which decreased delay repolarization in the myocardium and stabilized the ventricular substrate were comparable with previous investigations.

Previous studies have reported a prolongation of VERPs after RDN with similar VA inducibility^13,14^. In a model of HF, RDN significantly suppressed VA inducibility and VA burden^15^. Our study revealed comparable results with trends of VERP prolongation and significant reduction of VA inducibility. In addition, the administration of anesthetic agents was an important consideration during the experimental procedure^16,17^. It is suggested that the pentobarbital agents may potentially affect the circadian and cardiac rhythm leading to confounding results. However, an animal study has demonstrated the limited effects on repolarization and VA burden under anesthetic agents. The dissociation between the pharmacologic target of pentobarbital and the CV physiological actions was identified in a mice study. Therefore, the use of pentobarbital revealed a stable VA threshold and was considered one of the suitable models for cardiac EP studies.

In LQTS patients, an elevated sympathetic tone may trigger ventricular repolarization leading to fatal VAs, therefore, antiadrenergic agents have been the main goal for LQTS treatment. Despite the promising results from antiadrenergic agents, not all LQTS subtypes benefit from the current treatment, thus alternative therapy may offer additional insights on LQTS treatment. A previous study demonstrated renal sympathetic stimulation can increase both the systemic and cardiac sympathetic tone leading to potential VAs and SCD, whereas the RDN showed cardiac protective effects by stabilizing the ventricular substrate through decreased sympathetic nerve density^18^. Moreover, Chen et al. reported a positive correlation between sympathetic nerve density and fatal VAs^18^. The underlying mechanism of RDN includes the reduced secretion of norepinephrine (NE) by more than 80% after the RDN procedure^18,19^. In our previous research, the RDN significantly reduced the NE and epinephrine levels in the renal parenchymal tissues leading to the downregulation of sympathetic activity^10,15^. Moreover, the RDN results in significant stellate ganglion remodeling and influences afferent nerve fibers of the ganglia in the brainstem^18,20^.

In our study, the incidence of VA inducibility was lower significantly in all 3 subtypes of LQT-RDN groups than those in the LQT groups. Interestingly, our study showed results of reduced VA burden through RDN in the LQT3-RDN group sheds new light on the therapeutic strategies. In LQT3 subjects, the role of sympathetic activation in VA events is controversial and the efficacy of antiadrenergic agents on LQT3 subjects is uncertain. Wilde et al. demonstrated the benefits of RDN which attenuated QTc interval prolongation but prevented bradycardia in LQT3 subjects^21^. Our study revealed similar results in which the LQT3 group showed a lower VA burden and the SR CL prolongation was less prominent in comparison with the LQT1 and LQT2 groups. Importantly, the RDN in the LQT3-RDN group successfully decreased VA burden without affecting the heart rate which could be a potential substitute for an antiadrenergic agent in LQT3 subjects.

In this study, the major limitation was the different physiological and autonomic mechanisms between the rabbit and humans. An LQTS rabbit model induced by drugs cannot fully represent the real-world clinical conditions of congenital LQTS patients^22^. Second, the telemetric tools were not included in this study. Our study investigated the LQTS animal model induced by drugs, therefore it is not able to have telemetric monitoring in an ambulatory animal. However, previous investigations have proven the elevated sympathetic tone leading to increased VA inducibility which was compatible with our findings^23,24^. Third, our study was performed under GA rather than a conscious state which could potentially affect the results. However, an LQTS animal study and our previous RDN studies showed favorable results demonstrating the role of RDN^15,25,26^. Finally, autonomic blockades were not applied in this study to evaluate the intrinsic autonomic tone. Previous studies have demonstrated the RDN approach attenuated the sympathetic tone via elimination of peri-renal sympathetic neurons leading to reduced production of catecholamine levels^27^. The efficacy of RDN in the heart was demonstrated in our previous studies, in which the RDN led to a decreased density of sympathetic neurons of the myocardium and attenuated VA inducibility^10,15,26^. These studies have fully demonstrated the interaction between cardiac sympathetic tone and the RDN approach.

In summary, our findings suggested that RDN affects the sympathetic activity leading to QTc interval prolongation and reduction of VA burden in all the subtypes of LQTS models. These results offer additional insights on the characteristics of neuromodulation through RDN and highlight the potential benefits of RDN as an alternative antiadrenergic treatment in LQTS.

## Methods

### Animal preparation and LQTS model

The present study protocol was reviewed and approved by the Institutional Animal Care and Committee of Taipei Veterans General Hospital (IACUC: 2017-245). All authors in this study complied with the ARRIVE guidelines and confirmed all experiments were performed in accordance with the relevant guidelines and regulations. Drug induction of HMR-1556 (Bio-Techne Inc., USA), erythromycin (Famar L'Aigle Inc., France), and veratridine (Tocris Bioscience Inc., USA) were applied on rabbits during the final experimental procedure to mimic LQTS type 1 (LQT1), LQTS type 2 (LQT2), and LQTS type 3 (LQT3), respectively. The HMR-1556 is a slow component of delayed rectifier potassium (K^+^) current (IKs) inhibitor that inhibits the outward K^+^ current for myocardial repolarization. The erythromycin is a rapid component of delayed rectifier K^+^ current (IKr) inhibitor that inhibits the outward K^+^ current for myocardial repolarization. The veratridine is an inhibitor of sodium (Na^2+^) channel inactivation that results in the opening of the Na^2+^ channel during myocardial depolarization. Previous studies have proven the validity of the LQTS rabbit model induced by drugs selected in this study^28^. Detailed LQTS subtype induction and doses are described in the experimental procedure section. A total of 44 male New Zealand white rabbits (weight 2.5–3.0 kg, from Shulin Breeding facility, New Taipei, Taiwan) at 12 weeks of age were randomized into 6 groups of LQT1 (n = 8), LQT1-RDN (n = 7), LQT2 (n = 8), LQT2-RDN (n = 7), LQT3 (n = 7), and LQT3-RDN (n = 7). All rabbits underwent 2 anesthetic procedures, the first procedure was under intraperitoneal and intravenous anesthesia for RDN or sham (exposure of the abdominal cavity without RDN), and the second procedure was under general anesthesia (GA for the final experiment. In the first anesthetic procedure, the LQT1, LQT2, and LQT3 groups underwent surgical opening followed by the closure of the abdominal cavity without RDN, whereas the LQT1-RDN, LQT2-RDN, and LQT3-RDN groups underwent surgical and chemical RDN. After 4 weeks, all rabbits underwent the drug application to mimic LQTS for the final experimental procedure.

### Surgical and chemical RDN

The RDN procedure was performed 1 month before the experimental procedure. We selected a combination of surgical and chemical RDNs. This technique has been described in our previous publications with a confirmed RDN effect^15,26^. In brief, each rabbit underwent fasting for 1 night before the surgery. All rabbits were anesthetized with induction by intraperitoneal injection of sodium pentobarbital (40 mg/kg) and maintained by intravenous injection of xylazine (1 mg). The renal arteries were approached through a mid-abdominal incision. Under surgical RDN, bilateral renal arteries were surgically denervated by cutting all visible nerves in the area of the renal hilus and by removing 1 cm of the adventitia from the renal artery. Under chemical RDN, the area was then moistened with a 20% phenol solution for 15 min (mins). After the procedure, the abdomen was closed layer by layer. All rabbits received antibiotic agents (gentamycin 5 mg/kg) immediately after the procedure. Pain-controlling agents (ibuprofen 2 mg/kg) were placed in their water for 3 consecutive days. The final experimental procedure was performed after 4 weeks of maturation.

### Experimental procedure

During preparation, a warming blanket was used to maintain the rabbit’s body temperature. All animals were anesthetized with an intraperitoneal injection of sodium pentobarbital (40 mg/kg) followed by endotracheal intubated with ventilation. Inhalation of isoflurane 2% was administrated for maintaining GA. An intravenous (IV) line was set up for the medication infusion and fluid supplement. Thoracotomy was performed after subcutaneous injection of 5 ml lidocaine 1% (10 mg/ml). After local anesthesia, a pericardial incision was performed to expose the epicardial surface.

Electrocardiogram (ECG) measurements and cardiac EP study were performed in all rabbits. The ECG signals were amplified by a standard amplifier (Lab System TM PRO EP Recording System, Bard, MA, USA, filter setting:0.1–150 Hz). The QT interval and corrected QT interval (QTc) (Bazett’s formula) were measured at baseline and in each concentration of drug infusion.

Drug infusion of HMR-1556, erythromycin, and veratridine were administrated to mimic the acute onset of LQT1, LQT2, and LQT3, respectively. In the LQT1 and LQT1-RDN groups, HMR-1556 was administered in 4 sequentially increased drug doses to mimic the LQT1 syndrome. The initial IV infusion rate of HMR-1556 was 0.005 mg/kg/min for 30 min followed by an increased infusion rate of 0.01, 0.02, and 0.04 mg/kg/min for 30 min sequentially. In the LQT2 and LQT2-RDN groups, erythromycin was administered in 3 sequentially increased drug doses to mimic the LQT2 syndrome. The initial IV infusion rate of erythromycin was 133 nmol/kg/min for 30 min followed by an increased infusion rate of 266 and 400 nmol/kg/min for 30 min sequentially. In the LQT3 and LQT3-RDN groups, veratridine was administered in 4 sequentially increased drug doses to mimic the LQT3 syndrome. The initial IV infusion rate of veratridine was 0.5 µM/kg/min for 30 min followed by an increased infusion rate of 1, 2, and 4 µM/kg/min for 30 min sequentially. Cardiac EP study and VA inducibility test was done for all drugs under each concentration.

Cardiac EP study was performed by a custom-made stimulator (Model 5325, Medtronic, Ltd, Minneapolis, USA) that delivered constant-current pulses of 1 ms duration. The test was performed at baseline and during each concentration of drug infusion in all groups. Programmed electrical stimulation was delivered to the multielectrode catheter distal tips at 2 and 10 times the pacing thresholds at the RV and LV. Eight consecutive stimuli (S1S1 = 300 ms cycles) followed by a premature stimulus (S1S2). The S1S2 was initially decreased from 200 ms by decrements of 10 ms and then 1 ms when achieving an ERP, respectively. The VA inducibility test was performed by burst S1S1 ventricular pacing (cycle length decreasing from 250 ms to failure of 1:1 ventricular capture) was delivered to induce VAs. A positive VA inducibility test was defined as an incidence of sustained (> 30 secs) VA induced by the shortest ventricular pacing at 1:1 cycle length (usually between 100 and 150 ms, burst S1S1 pacing for 10 secs), with a maximum pacing output (10 mA) during 10 inductions^6^. The percentage of VA inducibility was presented by calculating the number of inducible sustained VAs in a total of 10 electrical inductions. If the VA was sustained for more than 30 secs, external electrical defibrillation was delivered to restore to SR. After the experimental procedure, euthanasia was performed in all rabbits by exsanguination during anesthesia.

### Statistical analysis

Continuous data were reported as mean value ± standard error. Categorical data were presented as absolute values and percentages. The difference between the 2 groups was compared by the Mann–Whitney test with a *p* value of < 0.05 was considered statistically significant. Analysis was performed by using SPSS statistical software (Version 20, SPSS Institute Inc, Chicago, IL, USA).

## Data Availability

The datasets generated during and/or analyzed during the current study are not publicly available due to confidential reasons but are available from the corresponding author on reasonable request.

## Abbreviations

CL: Cycle length
EP: Electrophysiology
ERP: Effective refractory period
GA: General anesthesia
ICD: Implanted cardioverter defibrillator
LCSD: Left cardiac sympathetic denervation
LQT: Long QT
LQT1: Long QT syndrome type 1
LQT2: Long QT syndrome type 2
LQT3: Long QT syndrome type 3
LQT-RDN: Long QT with renal artery denervation
LQTS: Long QT syndrome
LV: Left ventricle
QTc: Corrected QT interval
RDN: Renal artery denervation
RV: Right ventricle
SR: Sinus rhythm
VA: Ventricular arrhythmia
VERP: Ventricular effective refractory period
